# Regulation of feeding behavior and psychomotor activity by corticotropin-releasing hormone (CRH) in fish

**DOI:** 10.3389/fnins.2013.00091

**Published:** 2013-05-30

**Authors:** Kouhei Matsuda

**Affiliations:** Laboratory of Regulatory Biology, Graduate School of Science and Engineering, Graduate School of Innovative Life Science, University of ToyamaToyama, Japan

**Keywords:** goldfish, CRH, ICV injection, food intake, anorexigenic action, psychomotor activity, anxiogenic-like action

## Abstract

Corticotropin-releasing hormone (CRH) is a hypothalamic neuropeptide belonging to a family of neuropeptides that includes urocortins, urotensin I, and sauvagine in vertebrates. CRH and urocortin act as anorexigenic factors for satiety regulation in fish. In a goldfish model, intracerebroventricular (ICV) administration of CRH has been shown to affect not only food intake, but also locomotor and psychomotor activities. In particular, CRH elicits anxiety-like behavior as an anxiogenic neuropeptide in goldfish, as is the case in rodents. This paper reviews current knowledge of CRH and its related peptides derived from studies of teleost fish, as representative non-mammals, focusing particularly on the role of the CRH system, and examines its significance from a comparative viewpoint.

## Introduction

Corticotropin-releasing hormone (CRH), a 41-amino-acid neuropeptide present in the brains of vertebrates, was first isolated and characterized from the ovine hypothalamus (Vale et al., 1981), and then subsequently identified in non-mammalian brains (Lovejoy and Balment, 1999). CRH is a member of a family of related peptides that includes urotensin-I (UI), sauvagine, and urocortin/stresscopin in vertebrates (Lovejoy and Balment, 1999; Boorse and Denver, 2006). In mammals, CRH is known to induce the release of adenohypophyseal hormones such as adrenocorticotropic hormone (ACTH), β-endorphin, and α-melanocyte-stimulating hormone (α-MSH) from the pituitary, and there is ample evidence that CRH and its related peptides play multiple roles in animal development and also in physiological and behavioral adaptation to environmental changes and energy balance (Tonon et al., 1986; Hauger et al., 1988, 2006; Lowry and Moore, 2006; Cooper and Huhman, 2007; Denver, 2009; Papadimitriou and Priftis, 2009; Chen et al., 2012; Kubota et al., 2012).

In non-mammalian vertebrates such as amphibians and teleosts, CRH acts as a potent stimulator of corticotropin, thyrotropin, and α-MSH release (Boorse and Denver, 2004, 2006; Calle et al., 2005; Ito et al., 2006; Okada et al., 2007). CRH and its related peptides also act as regulators of feeding behavior and stress responses in vertebrates including mammals, birds, amphibians, and fish (Kalra et al., 1999; Bernier and Peter, 2001; Ohgushi et al., 2001; Hillebrand et al., 2002; Tachibana et al., 2004; Saito et al., 2005; Lowry and Moore, 2006; Carr et al., 2010; Matsuda et al., 2010b; Morimoto et al., 2011; Khan et al., 2013). It has been reported that, in the goldfish, intracerebroventricular (ICV) administration of CRH or UI exerts an anorexigenic action (de Pedro et al., 1997; Bernier and Peter, 2001; Volkoff et al., 2005; Matsuda, 2009), which is blocked by treatment with a CRH 1/CRH 2 receptor antagonist, α-helical CRH_(9−41)_ (de Pedro et al., 1997; Bernier and Peter, 2001; Bernier, 2006; Maruyama et al., 2006). In fish, ICV administration of CRH also affects locomotor activity (Clements and Schreck, 2004; Maruyama et al., 2006; Carpenter et al., 2007; Backström et al., 2011a; Ghisleni et al., 2012; Matsuda et al., 2013b), suggesting that CRH exerts psychophysiological effects in fish. Recent reports indicate that a fish's swimming pattern can be used to evaluate psychomotor activities, notably anxiety-like behavior (Faganello and Mattioli, 2007; Grossman et al., 2010; Maximino et al., 2010a,b; Matsuda et al., 2011a,b, 2013b; Blaser and Rosemberg, 2012; Maaswinkel et al., 2012). Therefore, the present mini-review summarizes recent advances in knowledge about the regulation of feeding behavior and locomotor or psychomotor activity by CRH and its related peptides in fish, especially with reference to the goldfish model.

## Control of food intake by CRH and its related peptides in fish

The effects of ICV administration of neuropeptides on food intake in goldfish have been extensively studied. For example, ICV-injected ghrelin, neuropeptide Y, and orexin increase food consumption whereas CRH, UI, proopiomelanocortin (POMC)-derived peptides such as α-MSH, pituitary adenylate cyclase-activating polypeptide (PACAP), cholecystokinin (CCK), neuromedin U (NMU), and diazepam-binding inhibitor-derived peptides such as octadecaneuropeptide (ODN) decrease food intake (Matsuda, 2009). These neuropeptides are not independently involved in the control of feeding behavior, but mutually interact with each other. The anorexigenic actions of PACAP and NMU are abolished by treatment with α-helical CRH_(9−41)_, and CCK- and ODN-evoked anorexigenic actions are also attenuated by treatment with the melanocortin 4 receptor (MC4R) antagonist HS024 (Maruyama et al., 2006, 2009; Kang et al., 2010; Matsuda et al., 2010a). These findings suggest that CRH and α-MSH mediate the actions of PACAP and NMU, and CCK and ODN, respectively. In goldfish, α-MSH-containing nerve fibers or endings lie in close apposition to CRH-containing neurons in a specific region of the hypothalamus, the nucleus posterioris periventricularis (NPPv). The anorexigenic action of the α-MSH agonist melanotan II (MT II) is abolished by treatment with α-helical CRH_(9−41)_ whereas the anorexigenic action of CRH is not affected by treatment with HS024 (Matsuda et al., 2008a). These observations indicate that, in goldfish, α-MSH-induced anorexigenic action is mediated by the CRH-signaling pathway, and that CRH plays a crucial role in the regulation of feeding behavior as an integrated anorexigenic neuropeptide in this species.

The distribution of CRH in the brain of teleost fish including the goldfish, has been well-reported: CRH-containing neuronal cell bodies are localized in various hypothalamic regions, including the preopticus periventricularis (NPP), the nucleus preopticus (NPO), the lateral part of the nucleus lateralis tuberis (NLTl) and the NPPv, and CRH-containing fibers or endings are distributed throughout the brain, and in the neurohypophysis (Olivereau et al., 1984, 1988; Yulis et al., 1986; Yulis and Lederis, 1987). For example, in goldfish, neuronal cell bodies exhibiting CRH-like immunoreactivity are located mainly in the preoptic parvocellular areas comprising the NPP and NPO, the NLTl, and paraventricular organ areas such as the NPPv, and their fibers are distributed in the diencephalon, mesencephalon, and neurohypophysis. CRH-containing neurons that originate in the NPP and NPO parvocellular population seem to innervate the pituitary. As described above, studies of the effect of CRH on feeding behavior in goldfish have shown that it acts as a powerful hypothalamic anorexigenic peptide (de Pedro et al., 1993, 1997; Bernier et al., 1999, 2004; Bernier and Peter, 2001; Maruyama et al., 2006). Interestingly, we and others have found that ICV injection of gonadotropin-releasing hormone 2 (GnRH2, also known as chicken GnRH II) affects food consumption, and that GnRH2 decreases food intake (Hoskins et al., 2008; Matsuda et al., 2008b). Subsequently it has been indicated that the anorexigenic actions of CRH and α-MSH are blocked by treatment with the GnRH type I receptor antagonist Antide, suggesting that GnRH2 mediates the actions of other anorexigenic neuropeptides examined so far, and that GnRH2 acts as a key neuropeptide exerting satiety control (Kang et al., 2011).

## Psychophysiological effect of CRH in fish

**Figure 1:**
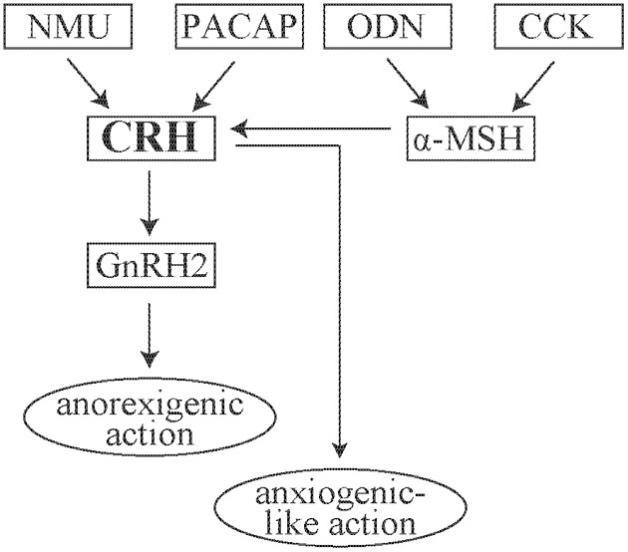
**Schematic drawings of the neuronal signaling pathways of anorexigenic and anxiogenic-like action in goldfish.** ODN and CCK-induced anorexigenic actions are mediated by α-MSH-signaling pathway, and the anorexigenic actions of NMU, PACAP, and α-MSH are mediated by CRH- and subsequent GnRH2-signaling pathways. CRH also evokes anxiogenic-like action. Abbreviations: NMU, neuromedin U; PACAP, pituitary adenylate cyclase-activating polypeptide; ODN, octadecaneuropeptide; CCK, cholecystokinin; CRH, corticotropin-releasing hormone; α-MSH, α-melanocyte-stimulating hormone; GnRH2, gonadotropin-releasing hormone 2.

Recent studies have shown that several neuropeptides such as CRH, GnRH2, ODN, PACAP, NPY, ghrelin, and orexin affect not only food intake but also locomotor activity in fish (Table 1): ICV injection of CRH enhances swimming distance, and stimulates locomotor activity (Maruyama et al., 2006; Carpenter et al., 2007; Backström et al., 2011a,b; Matsuda et al., 2013b). Psychophysiological compounds including diazepam, serotonin, a selective serotonin reuptake inhibitor Fluoxetin, a central-type benzodiazepine receptor inverse agonist FG-7142, and an N-methyl-d-aspartate receptor antagonist MK-801 also modify locomotor activity (Kang et al., 2010; Matsuda et al., 2011b, 2013b; Winder et al., 2012). Recent reports have indicated that the swimming pattern of a fish in a tank can be used to evaluate psychomotor activity (Faganello and Mattioli, 2007; Cachat et al., 2010; Grossman et al., 2010; Maximino et al., 2010a,b; Khor et al., 2011, 2013; Matsuda et al., 2011a; Piato et al., 2011). The scototaxis test (light/dark preference test) has been developed, and used for measuring psychomotor activity (Faganello and Mattioli, 2007; Blaser and Rosemberg, 2012). Intact animals usually prefer the dark area to the light area, and psychophysiological substances affect this preference: treatment with diazepam increases the time spent in the light area, and treatment with FG-7142 increases the time spent in the dark area, suggesting that the former and latter treatments induce anxiolytic- and anxiogenic-like actions, respectively (Matsuda et al., 2011b). Since intact goldfish and zebrafish prefer the lower to the upper area of a tank, another preference test has also been developed to evaluate the effect of CRH or other substances on psychomotor activity (Khor et al., 2013; Matsuda et al., 2013b). ICV administration of CRH and FG-7142 both increase the time taken to move from the lower to the upper area, and the anxiogenic-like action of CRH is blocked by treatment with α-helical CRH_(9−41)_ (Matsuda et al., 2013b). Recent studies of other fish have also indicated that CRH induces behavioral changes including anxiety and suppression of aggressive behavior (Lastein et al., 2008; Carpenter et al., 2009; Backström et al., 2011a,b; Ghisleni et al., 2012). These studies suggest that CRH exerts psychophysiological effects as an anxiogenic factor in addition to satiety control in fish. Figure 1 shows a schematic drawing of the anorexigenic signaling pathways mediated by CRH and other neuropeptides in the central nervous system of goldfish. As described above, CRH also evokes anxiogenic-like action in this species. Although it is unclear why regulation of food intake and the psychophysiological effects of CRH are closely linked, CRH appears to induce both anorexigenic- and anxiogenic-like actions in fish. Therefore, it is reasonable to suggest that the increased locomotor activity of fish in an experimental tank induced by CRH can be interpreted as escape behavior triggered by the anxiogenic-like action of CRH and subsequent stress response. Further study is warranted to clarify the function of CRH and its related peptides in the regulation of feeding and emotional activity in fish.

**Table 1:** **Effects of neuropeptides and psychophysiological compounds on food intake, locomotor activity, and emotional action in fish**.

**Substances**	**Species**	**Food intake**	**Locomotor activity**	**Emotional action**	**References**
CRH	Goldfish	Down	Up	Anxiogenic-like	Maruyama et al., 2006; Matsuda et al., 2013b
	Rainbow trout		Up	Anxiogenic-like	Carpenter et al., 2007; Backström et al., 2011a,b
GnRH2	Goldfish	Down	Up		Hoskins et al., 2008; Matsuda et al., 2008b
	Zebrafish	Down			Nishiguchi et al., 2012
ODN	Goldfish	Down	Up	Anxiogenic-like	Matsuda et al., 2007, 2011b
PACAP	Goldfish	Down	Up	Anxiogenic-like	Matsuda et al., 2006a, 2013a
NPY	Goldfish	Up	Down	Anxiolytic-like	Matsuda et al., 2011a, 2012b
	Zebrafish	Up			Yokobori et al., 2012
Ghrelin	Goldfish	Up	Up or Down		Matsuda et al., 2006b; Yahashi et al., 2012
ORX	Goldfish	Up	Up		Nakamachi et al., 2006; Matsuda et al., 2012a
	Zebrafish	Up	Up		Yokogawa et al., 2007; Yokobori et al., 2011
Diazepam	Goldfish		Down	Anxiolytic-like	Matsuda et al., 2011b
Fluoxetine	Sheepshead minnow		Down		Winder et al., 2012
	Chinook salmon		Down		Clements and Schreck, 2007
FG-7142	Goldfish		Up	Anxiogenic-like	Matsuda et al., 2011b
MK-801	Goldfish		Up		Kang et al., 2011

Abbreviations *CRH* *corticotropin-releasing hormone* *GnRH2* *gonadotropin-releasing hormone 2* *ODN* *octadecaneuropeptide* *PACAP* *pituitary adenylate cyclase-activating polypeptide* *NPY* *neuropeptide Y* *ORX* *orexin* *Fluoxetine* *a selective serotonin reuptake inhibitor* *FG-7142* *a central-type benzodiazepine receptor inverse agonist* *MK-801* *an N-methyl-d-aspartate receptor antagonist.*

## Conclusion

In fish, CRH exerts potential effects on food intake, as well as locomotor and psychomotor activities, providing an example of a neuropeptide that regulates both feeding behavior and psychophysiological activity such as anxiogenic- or anxiolytic-like action.

### Conflict of interest statement

The author declares that the research was conducted in the absence of any commercial or financial relationships that could be construed as a potential conflict of interest.
